# Molecular Evolution of the Deuterolysin (M35) Family Genes in *Coccidioides*

**DOI:** 10.1371/journal.pone.0031536

**Published:** 2012-02-20

**Authors:** Juan Li, Li Yu, Yanmei Tian, Ke-Qin Zhang

**Affiliations:** Laboratory for Conservation and Utilization of Bio-resources, and Key Laboratory for Microbial Resources of the Ministry of Education, Yunnan University, Kunming, People's Republic of China; University of Wyoming, United States of America

## Abstract

*Coccidioides* is a primary fungal pathogen of humans, causing life-threatening respiratory disease known as coccidioidomycosis (Valley fever) in immunocompromised individuals. Recently, Sharpton et al (2009) found that the deuterolysin (M35) family genes were significantly expanded in both the *Coccidioides* genus and in *U. reesii*, and that *Coccidioides* has acquired three more M35 family genes than *U. reesii*. In the present work, phylogenetic analyses based on a total of 28 M35 family genes using different alignments and tree-building methods consistently revealed five clades with high nodal supports. Interestingly, likelihood ratio tests suggested significant differences in selective pressure on the ancestral lineage of three additional duplicated M35 family genes from *Coccidioides* species compared to the other lineages in the phylogeny, which may be associated with novel functional adaptations of M35 family genes in the *Coccidioides* species, e.g., recent pathogenesis acquisition. Our study adds to the expanding view of M35 family gene evolution and functions as well as establishes a theoretical foundation for future experimental investigations.

## Introduction

*Coccidioides*, a type of dimorphic fungi in the order Onygenales, is composed of two closely related species, *Coccidioides posadasii* and *Coccidioides immitis* [1], [2]. They are primary fungal pathogens of humans, which can cause life-threatening respiratory disease known as coccidioidomycosis (Valley fever) in the immunocompromised individuals [3]–[6]. They infect about 150,000 people annually in the United States [4], and have been listed as one of the “U.S Health and Human Services Select Agents” of bioterrorism [7]. Such a designation has fueled research efforts to develop an effective human vaccine and new treatments against coccidioidomycosis [8], [9].

Among human pathogens, metalloproteinase (Mep) is one of the best known proteolytic enzymes playing important roles as key virulence factors in the patho-physiology of numerous human diseases [10]–[14]. Up to now, ten *Mep* genes in total (designated as *Mep1* to *Mep10*) have been identified in *C. posadasii* and they are classified into three families: pappalysin-1 metalloprotease (M43B), deuterolysin (M35), and metalloprotease (M36) families [14], [15]. Remarkably, by comparing the genome sequences of *Coccidioides* species and *U. reesii*, Sharpton et al [16] found that the M35 family genes, i.e., *Mep2* to *Mep8*, experienced gene duplication events before the divergence of *Coccidioides* genus and *U. reesii*, and moreover, *Coccidioides* acquired three additional *Mep* genes (*Mep2-like*, *Mep7-like* and *Mep8-like*) after it diverged from *U. reesii* [16].

Gene duplication has long been thought as a main event for evolutionary innovations and functional adaptations [17]–[21]. Up to now, several studies have revealed evidence for positive selection following gene duplication, leading to the emergence of novel functions (known as neofunctionalization) [18], [22]–[24]. Thus, the characterization of the molecular evolution of the M35 family genes in *Coccidioides* and *U. reesii* can be of great importance for understanding what evolutionary force has possibly shaped the diversification of these *Mep* genes in *Coccidioides* and *U. reesii*, and for inferring their biological significance.

## Materials and Methods

### Data sets

The genome sequences of *C. immitis* (AAEC02000000), *C. posadasii* (ACFW00000000), *Uncinocarpus reesii* (AAIW01000000), and other six fungal species with publically available genome sequences, including *Histoplasma capsulatum* (AAJI01000000), *Blastomyces dermatitidis* (ACBU00000000), *Paracoccidioides brasiliensis* (ABKI00000000), *Fusarium graminearum* f. sp. lycopersici (AAXH00000000), *Neurospora crassa* (AABX00000000), and *Phaeosphaeria nodorum* (AAGI00000000) were downloaded from GenBank database.

To find putative homologs of M35 family sequences from these nine genome sequences, we used the program HMMSEARCH from the HMMER package (http://hmmer.wustl.edu/) for homologous protein search, with the HMM profile Peptidase_M35 (PF02102; http://pfam.sanger.ac.uk/family/PF02102#tabview=tab6) used as query. Hits were considered significant when they matched the Pfam HMM profile with E values<10^−5^. In total, 28 M35 family genes were identified. In addition, we also used InterProScan (http://www.ebi.ac.uk/Tools/InterProScan/) [25] to analyze the domain compositions of all the 28 M 35 family genes.

### Sequence alignment

**Figure 1:**
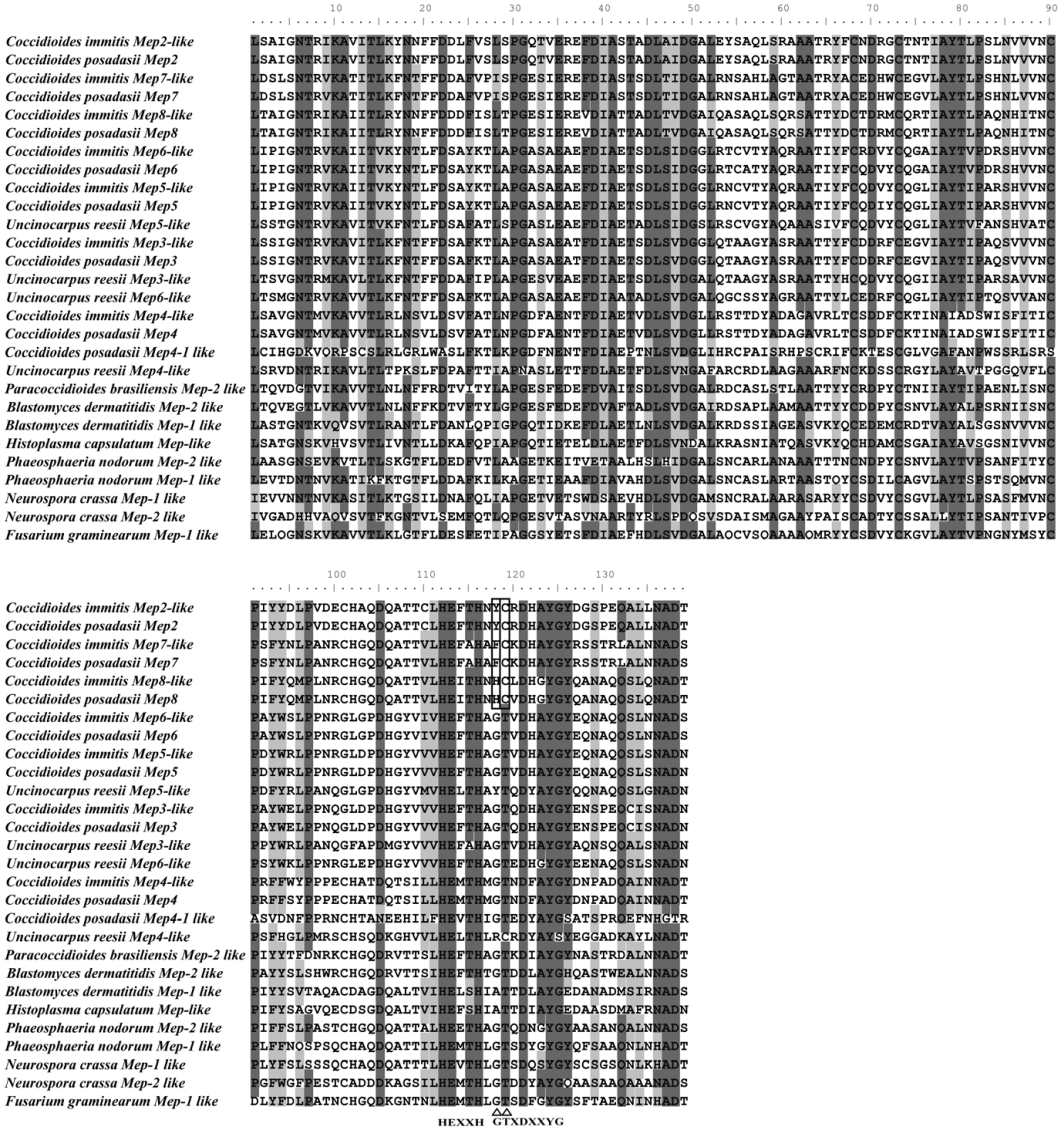
Protein alignment of 28 M35 genes. Areas shaded in black are conserved regions (100% similarity). Areas shaded in grey have a high degree of homology (more than 75% similarity), while those unshaded areas are highly variable regions between the proteases. Two positively selected residues identified in branch *i* are indicated.

To avoid the influence of a specific alignment program on the results, we applied three methods to yield alignments for analyses. In the first, MUSCLE v3.5 was first used to generate protein alignment with default settings [26]. Based on this protein alignment, PAL2NAL v13 was used to build a codon-based alignment with default settings [27]. A 417-bp codon-based alignment obtained from PAL2NAL v13 was shown in Figure 1 and the initial protein alignment generated by MUSCLE 3.5 that includes the trimmed sites was supplied (Fig. S1). In the second, sequences were aligned using the MUSCLE v3.5 software with default settings [26]. The ambiguous areas of alignment were located and removed by using the program Gblocks 0.91b [28], [29] with default parameters. The gap selection criterion “with half” was used here. A 918-bp alignment was shown in Fig. S2. In the third approach, sequences were aligned using PRANK with default settings [30], [31]. The ambiguous areas of alignment were located and removed by using the program Gblocks 0.91b [28], [29] with default parameters. The gap selection criterion “with half” was again used here. A 531-bp alignment was shown in Fig. S3.

### Phylogenetic analysis

Phylogenetic analyses of the alignments were performed using MEGA 5 [32] for Neighbor-joining (NJ) analysis, using PHYML 3.0 [33] for Maximum likelihood (ML) analysis, and using MrBayes 3.1.2 [34] for Bayesian inference. In the NJ analysis, Kimura-2 parameter model and pairwise deletion option for gaps were used. In the ML analysis, the model HKY+I+G of sequence evolution was optimized using Akaike information criterion [35], [36] as implemented in Modeltest version 3.7 [37]. The reliability of these tree topologies was evaluated using bootstrap support [38] with 1000 replicates for NJ and 100 for ML analysis.

The parameters estimated by Modeltest were also used in the priors of Bayesian inference with MrBayes version 3.1.2 [34]. Four Metropolis-coupled Markov chain Monte Carlo (MCMC) analyses were run for 2×10^5^ generations, sampling trees every 100 generations. The dataset was run for three times independently to avoid being trapped in local optimal. We determined the burn-in period by checking for likelihood stability. At the end of the run, the average standard deviation of split frequencies was less than 0.01 in all the cases, indicating a good convergence level (MrBayes 3.1.2 manual). A 50% majority rule consensus of post burn-in trees was constructed to summarize posterior probabilities (PPs) for each branch.

In addition, PhyloBayes 3.3b was run using the site-heterogeneous CAT model [39] with two independent Monte Carlo Markov Chain (MCMC) chains [40]. To check for convergence, the program bpcomp [41] was used to compare the bipartitions between the two runs. With a burn-in of 1000 and taking every two trees, the largest discrepancy (maxdiff) between the bipartitions was less than 0.1, indicating a good convergence level.

### Gene duplication and loss analyses

**Figure 2:**
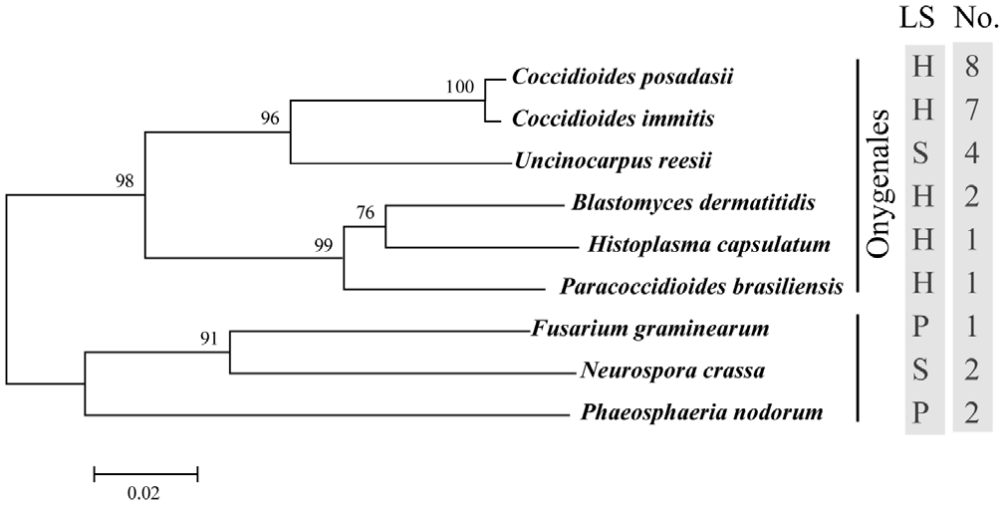
Species tree used for gene duplication and loss analyses in the study. The first column captures the life-styles of these nine fungi. H = human parasitic fungi; P = plant parasitic fungi; S = saprophytic fungi. The second column shows the gene numbers of M35 family gene in each fungus.

**Figure 3:**
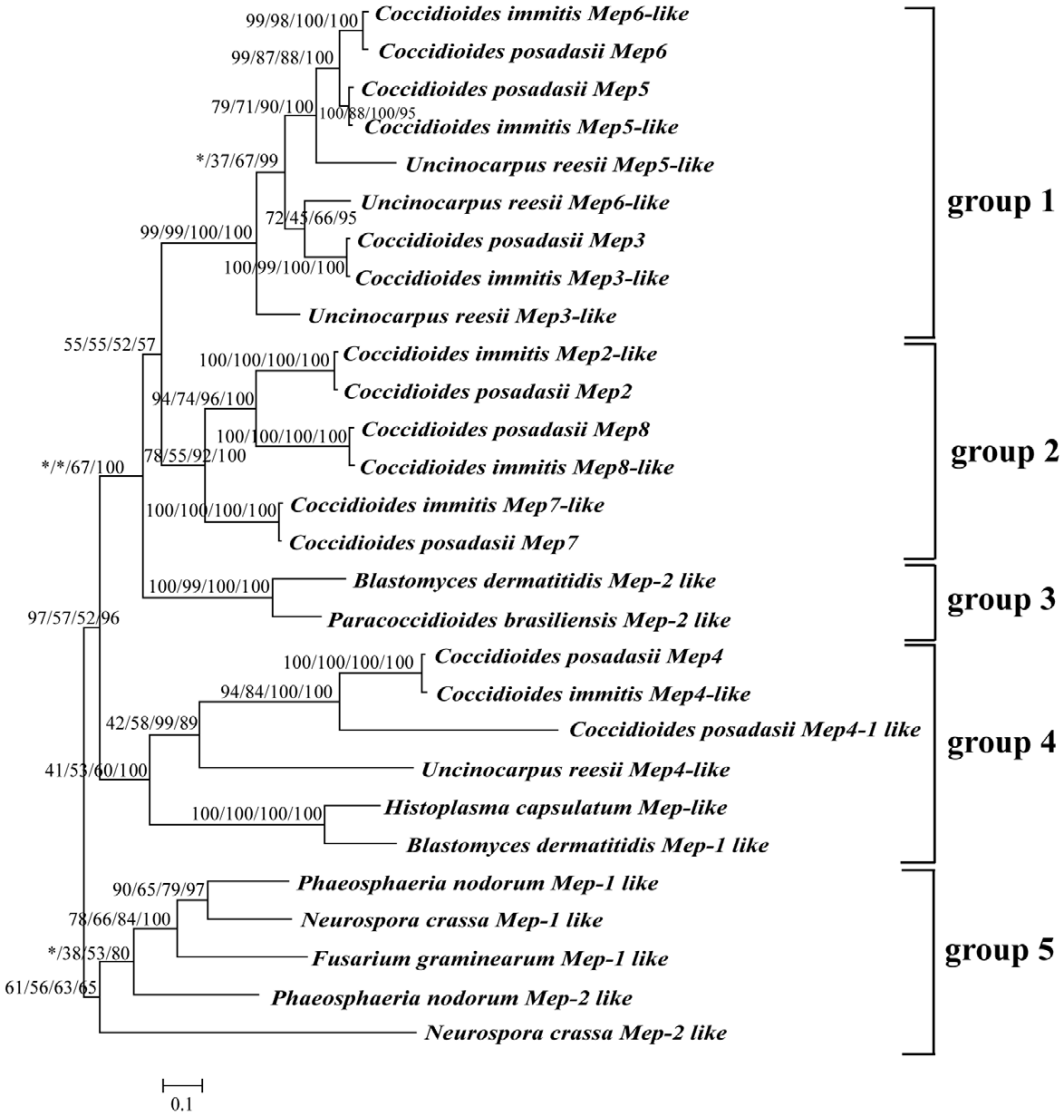
Phylogenetic tree based on 28 M35 family genes. Support values for the topology obtained from four analyses are listed as percentages in the order A/B/C/D. A is the bootstrap support from NJ analysis. B is the posterior probability from PhyloBayes. C is the bootstrap from ML analysis and D is the posterior probability from MrBayes. The symbol (*) indicates the topological differences between different trees.

**Figure 4:**
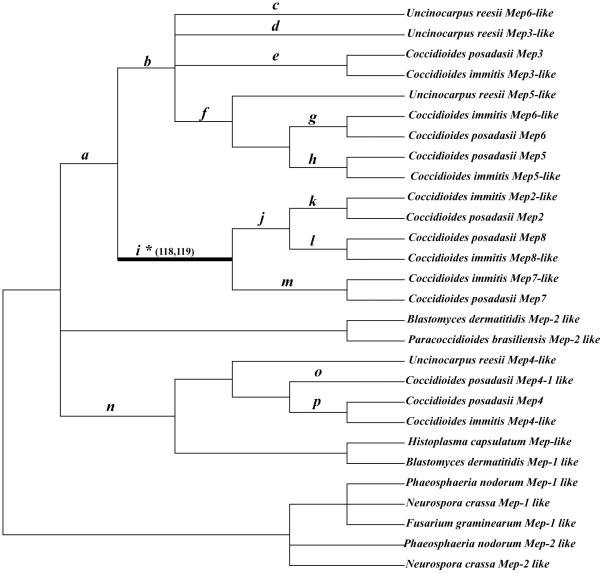
Phylogenetic tree of 28 M35 family genes used for codon-based maximum likelihood analysis in PAML. Phylogenetic trees were collapsed with inconsistent nodes from different tree-building methods and poor statistical supports into polytomy. Branches *a*–*p* indicated putative duplication events in *Coccidioides* species and *U. reesii*. The branch *i* with significant evidence of positive selection is indicated as a thick branch. Two positively selected residues predicted along this branch were also presented.

The reconciliation between species tree and gene tree along with the inferences of the gene duplication and loss scenarios were determined by Notung 2.6 [42], [43]. We infer the species tree by performing NJ analysis using MEGA 5 [32] from a combined alignment of six genes as those used in James et al [44], including 18S rRNA, 28S rRNA, ITS RNA, translation elongation factor 1-α (TEF1α), RNA polymerase II largest subunit (RPB1) and RNA polymerase II second largest subunit (RPB2) (Figure 2). For the gene tree used here, we collapsed those inconsistent nodes produced by different tree-building methods, which were also poorly supported into polytomy [45] (Figure 3 and Figure 4).

### Selective pressures analyses

The ratio ω (*d*_N_/*d*_S_) is the ratio of the number of non-synonymous substitutions per non-synonymous site (*d*_N_) to the number of synonymous substitutions per synonymous site (*d*_S_), which provides an indication of the change in selective pressures [46]. A *d*_N_/*d*_S_ ratio = 1, <1, and >1 are indicative of neutral evolution, purifying selection, and positive selection on the protein involved, respectively [47], [48].

To check whether there are substitution saturations in our data set, we plot the number of transitions and transversions vs. divergence using DAMBE, with an asymptotic relationship indicating the presence of saturation [49].

The codon substitution models implemented in the CODEML program in the PAML 4.4b package [50] were used to analyze changes of selective pressure. All models correct for transition/transversion rate and codon usage biases (F3×4). Different starting ω values were also used to avoid the local optima on the likelihood surface [51].

Two branch-specific models, i.e., “one-ratio” (M0) and “free-ratios”, were compared. M0 model assumes the same ω ratio for all branches while “free-ratios” model assumes an independent ω ratio for each branch [52]. We constructed likelihood ratio tests (LRT) to compare the two models. Significant differences between models were evaluated by calculating twice the log-likelihood difference following a χ^2^ distribution, with the number of degrees of freedom equal to the difference in the numbers of free parameters between models.

Site-specific models, which allow for variable selection patterns among amino acid sites, M1a, M2a, M7, and M8, were used to test for the presence of sites under positive selection and identify them. Significant differences between the 2 models were evaluated by calculating twice the log-likelihood difference following χ^2^ distribution, with the number of degrees of freedom equal to the difference in the numbers of free parameters between the 2 models. M2a and M8 models allow for positively selected sites. When these 2 positive-selection models fitted the data significantly better than the corresponding null models (M1a and M8a), the presence of sites with ω>1 would be suggested. The conservative Empirical Bayes approach [53] was then used to calculate the posterior probabilities of a specific codon site and identify those most likely to be under positive selection.

Considering that positive selection may act in very short episodes during the evolution of a protein [54] and affect only a few sites along a few lineages in the phylogeny, the likelihood models accommodating ω ratios to vary both among lineages of interest and amino acid sites, that are an improved version of the “branch-site” model, were also considered here [55]. We used branch-site Model A as a stringency test (test 2) and identified amino acid sites under positive selection by an empirical Bayes approach along the lineages of interest [55], [56]. The log-likelihoods for the null and alternative models were used to calculate a likelihood ratio test statistic, which was then compared against the χ2 distribution (with a critical value of 3.84 at a 5% significance level) [50]. In addition, the Bonferroni correction [57], [58] was also applied for multiple testing in the analysis according to the number of tests of significance performed.

### Homology Modeling

Using the 2.5 Å deuterolysin metalloproteinase from Aspergillus oryza (PDB ID: 1EB6A) [59], we conducted homology modeling with the SWISS-PROT program (http://swissmodel.expasy.org/) [60]–[62] and analyzed the structure with Pymol Ver. 0.99 [63] to investigate the possible functional shifts of the positively selected sites identified here.

## Results

### Characterizations of M35 family genes

As shown in Table 1, a total of 28 M35 family genes were identified from 9 fungal genome sequences. All these genes are consisted of only one *Mep* domain identified by InterProScan (Fig. S4). We used the Kimura 2-parameter model with MEGA 5 [32] to calculate the average genetic distance of M35 family genes, and found that the average genetic distance within this family was 0.640. In *C. immitis*, 7 genes were identified. In *C. posadasii*, 8 genes were identified, including 7 previously identified genes [14], and one gene (CPAT_05384) newly determined here (referred as *Mep4-1 lik*e gene). In addition, 4 genes were predicted from *U. reesii*. For the other six fungal species, one M35 family gene each was predicted from *H. capsulatum*, *P. brasiliensis* and *F. graminearum*, while two genes each were identified from *B. dermatitidis*, *N. crassa* and *P. odorum* (Table 1 and Figure 2).

**Table 1:** Characterization of M35 family genes from nine fungi we used.

Species name	Designated Gene name	Accession no.	Length(bp)	Intron	aa
*C. immitis*	*Mep2-like*	CIMG_07349T0	1062	2	353
	*Mep3-like*	CIMG_11800T0	1065	2	354
	*Mep4-like*	CIMG_00508T0	1083	2	360
	*Mep5-like*	CIMG_03010T0	1173	2	390
	*Mep6-like*	CIMG_05736T0	1209	2	402
	*Mep7-like*	CIMG_08613T0	1074	2	357
	*Mep8-like*	CIMG_10101T0	1065	2	354
*C. posadasii*	*Mep2*	CPAT_04742	1062	2	353
	*Mep3*	CPAT_04075	1065	2	354
	*Mep4*	CPAT_02396	1113	2	370
	*Mep5*	CPAT_08667	1173	2	390
	*Mep6*	CPAT_08585	1209	2	402
	*Mep7*	CPAT_05050	1059	2	352
	*Mep8*	CPAT_07671	1065	2	354
	*Mep4-1 like*	CPAT_05384	465	0	154
*U. reesii*	*Mep6-like*	URET_02006	1074	2	357
	*Mep3-like*	URET_03761	1071	2	356
	*Mep4-like*	URET_04198	972	2	323
	*Mep5-like*	URET_01255	1083	2	360
*H. capsulatum*	*Mep-1 like*	HCAG_05788T0	1113	2	370
*P. brasiliensis*	*Mep-2 like*	PADG_00776T0	1077	2	358
*B. dermatitidis*	*Mep-1 like*	BDCG_03454T0	1092	3	363
	*Mep-2 like*	BDCG_00922T0	1080	2	359
*F. graminearum*	*Mep-1 like*	FGST_09903	1143	2	380
*N. crassa*	*Mep-1 like*	Ncra_OR74A:NCU05071.t1	1056	2	351
	*Mep-2 like*	Ncra_OR74A:NCU05908.t1	1062	2	353
*P. nodorum*	*Mep-1 like*	Pnod_SN15:SNOG_02177.t1	1065	2	354
	*Mep-2 like*	Pnod_SN15:SNOG_10522.t1	1053	2	350

### Phylogenetic analysis

Phylogenetic analyses based on the 28 M35 family genes using different alignments and tree-building methods consistently recovered five clades (designated as Group 1 to 5; Figure 3, Fig. S5 and Fig. S6). Figure 3 shows the results based on the alignment strategy 1. Group 1 (all BS and PP≥99%) contained *Mep3-like*, *Mep5-like* and *Mep6-like* genes from *C. posadasii*, *C. immitis*, and *U. reesii*. It seemed that these genes were duplicated before the divergence of the three species. Group 2 (BS = 78–92%; PP = 55–100%) is a *Coccidioides*-specific lineage, containing three additional duplicated genes (*Mep2-like*, *Mep7-like* and *Mep8-like*) from each of the two *Coccidioides* species. They are most likely to have duplicated before the divergence of two *Coccidioides* species. Group 1 and Group 2 are more closely related to each other than to other groups. Group 3 (all BS and PP≥99%) is consisted of two *Mep2*-*like* genes from *B. dermatitidis* and *P. brasiliensis*. Group 4 (BS = 41–60%; PP = 53–100%) contained *Mep4-lik*e genes from both *Coccidioides* and *U. reesii*, and *Mep4-1 lik*e gene from *C. posadasii* as well as two *Mep-like* genes from *H. capsulatum* and *B. dermatitidis*. Group 5 (BS = 61–63%; PP = 56–65%) contained *Mep*-like genes from *F. graminearum*, *N. crassa* and *P. odorum*.

### Gene duplication and loss analysis

**Figure 5:**
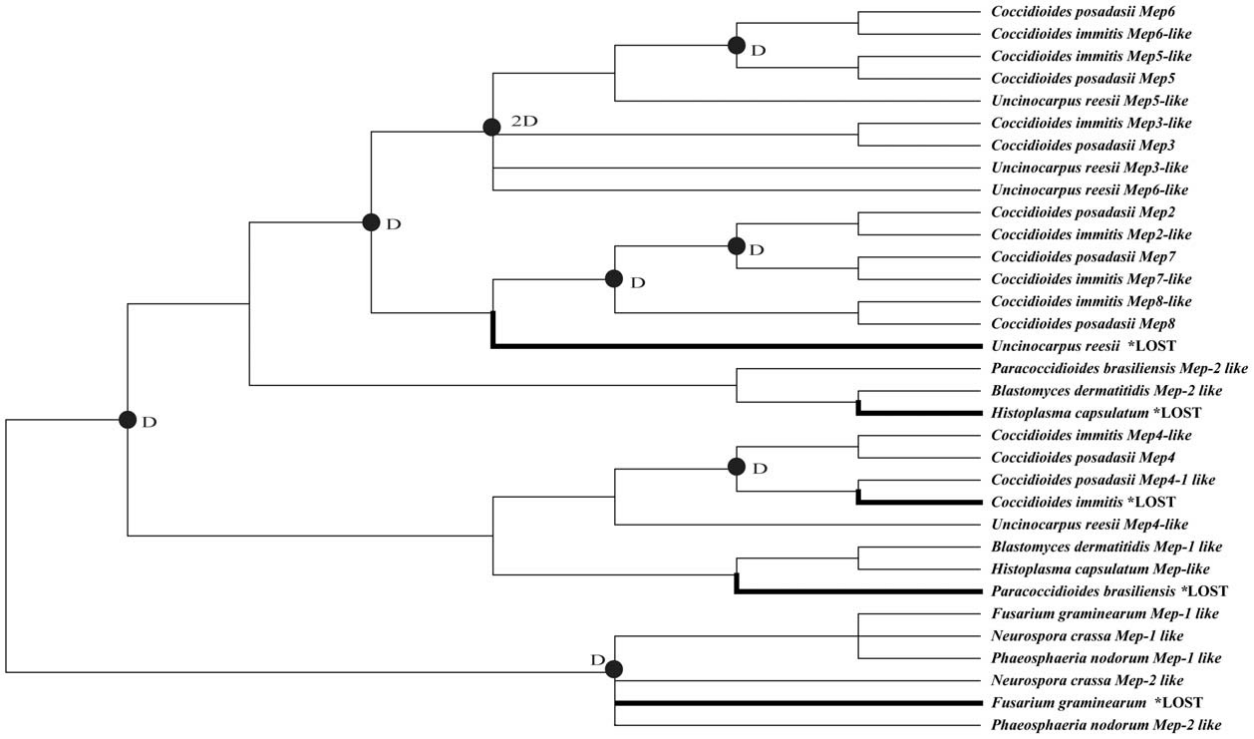
Duplication and loss events of M35 family genes. The reconciliation between species tree and gene tree along with the confirmation of the gene loss/duplication scenario were determined by using Notung 2.6 [43], [44]. The species tree is shown as Figure 1. The gene tree is shown as Figure 4. Putative duplication events are indicated with solid cycles, while loss events are indicated with thick branches. Two gene duplications were inferred at the ancestral lineage of Group 1 based on the no-binary topology.

The inferences of gene duplication and loss events are shown in Figure 5. We found that there were at least 9 gene duplication and 5 gene loss events during the evolutionary history of these M35 family genes and most of the gene duplication and loss events happened in the Onygenales species. An initial duplication event emerged in the ancestral lineage leading to Onygenales. Subsequently, three gene duplications occurred before the divergence of *Coccidioides* and *U. reesii*, and 4 gene duplications occurred before the divergence of *C. posadasii* and *C. immitis* (Figure 5). In comparison, one gene might have lost in *C. immitis*, *U. reesii*, *P. brasiliensis* and *H. capsulatum* each (Figure 5).

### Selective pressure analyses

The DAMBE analyses suggested that there was no evidence for substitution saturation in our data set (Fig. S7). Because the likelihood analysis might be sensitive to tree topology used, we collapsed the nodes that showed inconsistent branching patterns from different tree-building methods and with poor statistical support into polytomy [45] (Figure 4).

Table 2 shows the results of positive selection analyses inferred from alignment strategy 1. In the branch-specific model analyses [64], the ω ratios calculated in the one-ratio model (M0) is 0.11784, suggesting that most M35 family genes in these species have evolved under strong functional constraints. Interestingly, the free-ratio model shows a significantly better fit to the data than the M0 model (2ΔL = 109.5459, df = 49, p<0.001) (Table 2), indicating that these *Mep* genes have been the subjects of different selective pressures. A similar result was obtained based on alignment strategies 2 and 3 (see Table S1).

**Table 2:** CODEML analyses of selective pattern for M35 family genes.

Models		In*L* a	Parameter Estimates	2Δ*L* b	Positively Selected Sites^c^
Branch-specific models	M0	−8202.731187	ω = 0.11799	109.5459***	
	free-ratio	−8147.95822			
Site-specific models	M1a	−8061.584018	ω0 = 0.11403, ω1 = 1, p0 = 0.78978, p1 = 0.21022	0	Not allowed
	M2a	−8061.584018	ω0 = 0.11403, ω1 = 1,ω2 = 1, p0 = 0.78978,p1 = 0.12279, p2 = 0.08743		None
	M7	−7920.178512	p = 0.78018, q = 4.05964	0	Not allowed
	M8	−7920.178512	p = 0.98512, q = 6.50978, p0 = 0.93821, p1 = 0.6179, ω = 1.000		None
Branch-site models(Branch *i*)	Null	−8056.848368	ω0 = 0.11022, ω1 = 1, ω2 = 1, p0 = 0.65553,p2a = 0.13824, p2b = 0.03592	22.4986***	118(0.959), 119(0.997)
	Alterative	−8045.599058	ω0 = 0.11079, ω1 = 1, **ω2 = ∞**, p0 = 0.72865,p2a = 0.06967, p2b = 0.01760		

a In*L* is the log-likelihood scores.

b LRT to detect adaptive evolution.

***P<0.001.

c Posterior probability value of each codon site was showed in parentheses.

In the site-specific model analyses, both the positive-selection models (M2a and M8) did not provide a significantly better fit to the data than did the neutral models (M1a and M8a) (P = 1.000, respectively), A similar result was obtained based on alignment strategies 2 and 3 (see Table S1).

To investigate the possible selective forces behind the *Mep* gene duplication in *Coccidioides* species and *U. reesii*, we conducted LRTs based on the branch-site models for those branches resulted from gene duplications (16 branches in total, *a–p* as indicated in Figure 4). The analyses suggest that there are five branches (branches *a*, *d*, *i*, *j* and *l*) showing signs of positive selection (Figure 4). After Bonferroni correction for multiple testing, we found that LRT tests were still significant in branch *i*, leading to the common ancestor of 3 additional duplicated genes in *Coccidioides* (2ΔL = 22.4986, P = 0.0001) (Table 2 and Figure 4). As summarized in Table 2, the Bayesian approach in PAML predicted two sites located in mature peptide, G118Y/F/H and T119C, as positively selected for branch *i* with high BEB posterior probabilities larger than 0.95 (Figure 1and Table 2).

When we performed the branch-site model tests based on other two alignment strategies, we found that branch *i* consistently displayed significant signs of positive selection after the Bonferroni correction. In addition, G118Y/F/H and T119C were consistently predicted as positively selected sites along this branch. In summary, our results showed that positive selection is most likely to have at least acted on the lineages leading to the common ancestor of 3 additional duplicated genes in *Coccidioides.*

### Homology modeling

**Figure 6:**
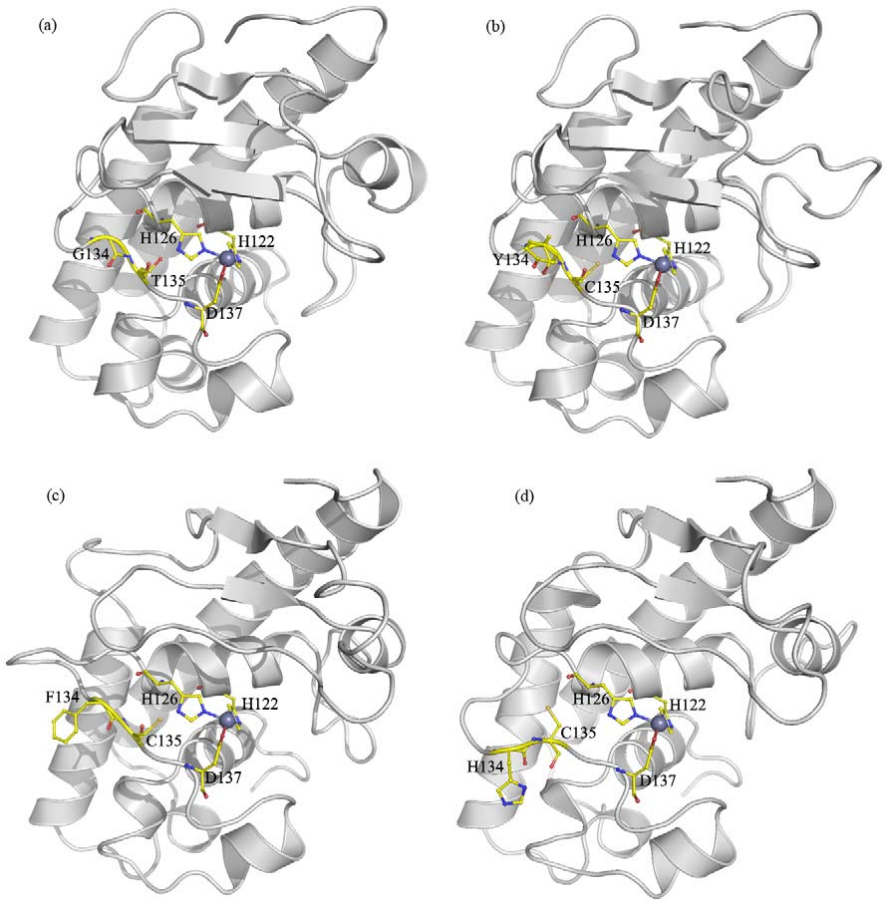
Homologous modeling of respective M35 family proteases in *Coccidioides*. (a)Mep 4; (b) Mep 2; (c) Mep7; (d) Mep8. The three catalytic zinc-binding residues and the two putative positive selected sites are shown as ball-and-stick models. Atoms are coloured with carbon in yellow, oxygen in red, nitrogen in blue and zinc in gray. All molecules are aligned in the same orientation.

Ninteen M35 family proteases from *Coccidioides* and *U. reesii* were modeled and evaluated to have good structural qualities (Table S2). We then mapped two sites, G118Y/F/H and T119C (corresponding to sites 134 and 135 of the model, respectively), that were consistently showed positively selected based on three different alignment strategies onto the structure models (Figure 6). Interestingly, all the three substituted amino acid residues, i.e., Tyrosine, Phenylalanine and Histidine, occurred in site 134 have larger side chains than Glycine in the other sequences. No obvious structure difference between T135 and C135 was identified from the structure models (Figure 6).

## Discussion

In the present paper, we analyzed the deuterolysin (M35) family genes in 9 Ascomycota species, adding the growing diversity of the *Mep* gene evolution. We found that the M35 family genes are significantly expanded in *Coccidioides* and *U. reesii* compared with the other fungal species examined. Phylogenetic reconstruction and gene duplication/loss analyses of these sequences placed the first gene duplication event in the common ancestor of the Onygenales species (Figure 3 and Figure 5). Subsequently, significantly more *Mep* genes were produced by additional gene duplication events in *Coccidioides* (7 genes in *C. immitis* and 8 genes in *C. posadasii*) and *U. reesii* (4 genes) compared with those in other Onygenales species examined (1 or 2 genes) (Figure 5). The observation of obvious expansion of M35 family genes in the present study in *Coccidioides* and *U. reesii* based on more Onygenales species supports earlier prediction of Sharpton et al [16].

Noticeably, we further showed that positive selection promoted this unusual gene expansion in *Coccidioides*, at least along the ancestral lineage producing the three additional duplicated genes specifically in *Coccidioides* species (Table 2 and Figure 4). As far as we know, this study is the first demonstration that positive selection has acted on the duplicated *Mep* genes during evolution. In addition, our study provided valuable information on the potentially important adaptive amino acid replacements. Among them, residues G118Y/F/H and T119C were consistently recovered as positively selected sites from the analysis of all three different alignment strategies with high posterior probabilities (>95%). For M35 family genes, the active zinc ligands are composed of 2 histidines in the HExxH motif and the Aspartic acid in motif GTXDXXYG [15]. Intriguingly, we found that these 2 putative positive selected sites were located within the motif GTXDXXYG. For site 118, the original amino acid G changed to Y (*Mep2*), F (*Mep7*) or H (*Mep8*) in the three additional duplicated Meps in *Coccidioides* species. Moreover, when we mapped them on the 3-dimensional crystal structure of the molecule, the substitutions that occurred at G118Y/F/H (corresponding to sites 134 in Figure 6) were predicted to have larger side chains. It is generally thought that side-chain of residues can influence the flexibility of the ligand-binding site of a protein [65], [66]. Therefore, these substitutions may exert influences on the binding of the zinc ion. For site 119 (corresponding to sites 135 in Figure 6), it has been considered important for M35 family gene because the hydroxyl group of T135 can interact with the second zinc binding histidine (H126), playing an important role in sustaining the coordination of the catalytic zinc ligands [15]. In our study, at site 135, T changed to C in all three additional duplicated proteases in *Coccidioides* species, indicating that this substitution may have influence on the coordination of the zinc ligands and protein flexibilities. (Figure 1 and Figure 6). Therefore, the two putative positively selected sites identified here may have profound effect on protein flexibility and zinc binding, which may lead to activity changes of the three additional duplicated proteases in *Coccidioides*.

Though the functional changes brought by these 2 positively selected residues cannot be predicted at present, they likely have functional consequences, raising the possibility that new physiologic functions of *Mep* genes have been developed in *Coccidioides*. Therefore, we proposed that the evidence of positive selection observed in the *Coccidioides* species might be associated with novel functional adaptations of M35 family genes. Presently, the experimental data concerning the expression of these paralogous M35 family genes in the *Coccidioides* is unavailable, so it is difficult to tell what selective pressure has promoted *Mep* gene expansion in this genus. One of the most likely speculations was that the presence of three additional *Mep* genes in *Coccidioides* was an adaptation to its recent pathogenesis acquisition because *Coccidioides* diverged from the nonpathogenic fungus *U reesii* and acquire its pathogenic phenotype relatively recently [16]. Of course, we can't exclude the possibility that these *Mep* genes in *Coccidioides* may serve other yet unknown physiological functions.

In conclusion, while novel information was generated through our analyses, more information on the structure, function, and evolution of M35 family genes is required to understand why significant expansion of *Mep* genes occured in *Coccidioides* and *U reesii*, and not in the other species. Our study established a foundation for the experimental investigations. It will be interesting to test the expression pattern of these *Mep* genes and the functional effects of amino acid substitutions for the identified positively selected sites.

## Supporting Information

Figure S1 **Whole protein alignment of the M35 genes with MUSCLE 3.5.** Sequences were aligned using MUSCLE v3.5 with default settings [26]. 139 amino acids (corresponding to 417-bp nucleotide positions) obtained from PAL2NAL v13 [27] were encompassed by frame with black edge.
(DOC)

Figure S2 **Protein alignment of M35 genes aligned with strategy 2.** Sequences were aligned using MUSCLE v3.5 software with default settings [26]. The ambiguous areas of alignment were located and removed by using the program Gblocks 0.91b [28], [29] with default parameters. The gap selection criterion “with half” was used here. A 918-bp alignment was obtained.
(DOC)

Figure S3 **Protein alignment of M35 genes aligned with strategy 3.** Sequences were aligned using PRANK with default settings [30], [31]. The ambiguous areas of alignment were located and removed by using the program Gblocks 0.91b [28], [29] with default parameters. The gap selection criterion “with half” was used here. A 531-bp alignment was obtained.
(DOC)

Figure S4 **Domain compositions of M35 family genes analyzed with InterProScan.** InterProScan (http://www.ebi.ac.uk/Tools/InterProScan/) [25] was used to analyze domain compositions of all the 28 M35 family genes.
(DOC)

Figure S5 **Phylogenetic trees of M35 family genes based on the alignment strategy 2.** Support values for the topology obtained from four analyses are listed as percentages in the order A/B/C/D. A is the bootstrap support from NJ analysis. B is the posterior probability from PhyloBayes. C is the bootstrap from ML analysis and D is the posterior probability from MrBayes. The symbol (*) indicates the topological differences between different trees.
(DOC)

Figure S6 **Phylogenetic trees of M35 family genes based on the alignment strategy 3.** Support values for the topology obtained from four analyses are listed as percentages in the order A/B/C/D. A is the bootstrap support from NJ analysis. B is the posterior probability from PhyloBayes. C is the bootstrap from ML analysis and D is the posterior probability from MrBayes. The symbol (*) indicates the topological differences between different trees.
(DOC)

Figure S7 **Plot of transitions/transversions versus genetic distance for M35 family genes.** The estimated number of transitions (s) and transversions (v) for each pairwise comparison is plotted against the genetic distance (d) calculated with the TN93 model of nucleotide substitution using DAMBE [49].
(DOC)

Table S1 **CODEML analyses of selective pressures for M35 family genes based on the alignment strategies 2 and 3.**
(XLS)

Table S2 **Homology modelings of M35 family proteases in** ***Coccidioides*** **and** ***U. reesii*** **.**
(XLS)

## References

[1] Saubolle MA, McKellar PP, Sussland D (2007). Epidemiologic, clinical, and diagnostic aspects of coccidioidomycosis.. Journal of Clinical Microbiology.

[2] Fisher M, Koenig G, White T, Taylor J (2002). Molecular and phenotypic description of *Coccidioides posadasii sp. nov.*, previously recognized as the non-California population of *Coccidioides immitis*.. Mycologia.

[3] Cole G, Xue JM, Okeke C, Tarcha E, Basrur V (2004). A vaccine against coccidioidomycosis is justified and attainable.. Medical mycology.

[4] Hector R, Laniado-Laborin R (2005). Coccidioidomycosis—a fungal disease of the Americas.. PLoS Medicine.

[5] Smith CE (1940). Epidemiology of acute coccidioidomycosis with erythema nodosum (“San Joaquin” or “Valley Fever”).. American Journal of Public Health.

[6] Galgiani JN (1999). Coccidioidomycosis: a regional disease of national importance: rethinking approaches for control.. Annals of Internal Medicine.

[7] Dixon D (2001). *Coccidioides immitis* as a select agent of bioterrorism.. Journal of Applied Microbiology.

[8] Hung CY, Yu JJ, Seshan KR, Reichard U, Cole GT (2002). A parasitic phase-specific adhesin of *Coccidioides immitis* contributes to the virulence of this respiratory fungal pathogen.. Infection and Immunity.

[9] Hector R, Rutherford GW (2007). The public health need and present status of a vaccine for the prevention of Coccidioidomycosis.. Annals of the New York Academy of Sciences.

[10] Creighton TE (1993). Proteins: Structures and molecular properties. 2nd Ed., ed.

[11] Vallee BL, Auld DS (1990). Zinc coordination, function, and structure of zinc enzymes and other proteins.. Biochemistry.

[12] Ramos O, Selistre-de-Araujo H (2001). Identification of metalloprotease gene families in sugarcane.. Genetics and Molecular Biology.

[13] Miyoshi S, Shinoda S (2000). Microbial metalloproteases and pathogenesis.. Microbes and infection.

[14] Hung C, Seshan K, Yu J, Schaller R, Xue J (2005). A metalloproteinase of *Coccidioides posadasii* contributes to evasion of host detection.. Infection and immunity.

[15] Hori T, Kumasaka T, Yamamoto M, Nonaka T, Tanaka N (2001). Structure of a newaspzincin'metalloendopeptidase from Grifola frondosa: implications for the catalytic mechanism and substrate specificity based on several different crystal forms.. Acta Crystallographica Section D: Biological Crystallography.

[16] Sharpton T, Stajich J, Rounsley S, Gardner M, Wortman J (2009). Comparative genomic analyses of the human fungal pathogens *Coccidioides* and their relatives.. Genome Research.

[17] Nowak MA, Boerlijst MC, Cooke J, Smith JM (1997). Evolution of genetic redundancy.. Nature.

[18] Lynch M, Conery JS (2000). The evolutionary fate and consequences of duplicate genes.. Science.

[19] Ohno S (1970). Evolution by gene duplication.

[20] Zhang J (2003). Evolution by gene duplication: an update.. Trends in Ecology and Evolution.

[21] Force A, Lynch M, Pickett FB, Amores A, Yan Y (1999). Preservation of duplicate genes by complementary, degenerative mutations.. Genetics.

[22] Loughran N, O'Connor B, Ó'Fágáin C, O'Connell M (2008). The phylogeny of the mammalian heme peroxidases and the evolution of their diverse functions.. BMC Evolutionary Biology.

[23] Chapman MA, Leebens-Mack JH, Burke JM (2008). Positive selection and expression divergence following gene duplication in the sunflower CYCLOIDEA gene family.. Molecular Biology and Evolution.

[24] Lynch M, O'Hely M, Walsh B, Force A (2001). The probability of preservation of a newly arisen gene duplicate.. Genetics.

[25] Zdobnov EM, Apweiler R (2001). InterProScan - an integration platform for the signature-recognition methods in InterPro.. Bioinformatics.

[26] Edgar R (2004). MUSCLE: multiple sequence alignment with high accuracy and high throughput.. Nucleic Acids Research.

[27] Suyama M, Torrents D, Bork P (2006). PAL2NAL: robust conversion of protein sequence alignments into the corresponding codon alignments.. Nucleic Acids Research.

[28] Castresana J (2000). Selection of conserved blocks from multiple alignments for their use in phylogenetic analysis.. Molecular Biology and Evolution.

[29] Talavera G, Castresana J (2007). Improvement of phylogenies after removing divergent and ambiguously aligned blocks from protein sequence alignments.. Systematic Biology.

[30] Löytynoja A, Goldman N (2005). An algorithm for progressive multiple alignment of sequences with insertions.. Proceedings of the National Academy of Sciences of the United States of America.

[31] Löytynoja A, Goldman N (2008). Phylogeny-aware gap placement prevents errors in sequence alignment and evolutionary analysis.. Science.

[32] Tamura K, Peterson D, Peterson N, Stecher G, Nei M (2011). MEGA5: molecular evolutionary genetics analysis using maximum likelihood, evolutionary distance, and maximum parsimony methods.. Molecular Biology and Evolution.

[33] Guindon S, Gascuel O (2003). A simple, fast, and accurate algorithm to estimate large phylogenies by maximum likelihood.. Systematic Biology.

[34] Ronquist F, Huelsenbeck J (2003). MrBayes 3: Bayesian phylogenetic inference under mixed models.. Bioinformatics.

[35] Akaike H (1974). A new look at the statistical model identification.. Automatic Control, IEEE Transactions on.

[36] Posada D, Buckley TR (2004). Model selection and model averaging in phylogenetics: advantages of Akaike information criterion and Bayesian approaches over likelihood ratio tests.. Systematic Biology.

[37] Posada D, Crandall KA (1998). MODELTEST: testing the model of DNA substitution.. Bioinformatics.

[38] Felsenstein J (1985). Confidence limits on phylogenies: an approach using the bootstrap.. Evolution.

[39] Lartillot N, Philippe H (2004). A Bayesian mixture model for across-site heterogeneities in the amino-acid replacement process.. Molecular Biology and Evolution.

[40] Lartillot N, Lepage T, Blanquart S (2009). PhyloBayes 3: a Bayesian software package for phylogenetic reconstruction and molecular dating.. Bioinformatics.

[41] Dutheil J, Boussau B (2008). Non-homogeneous models of sequence evolution in the Bio++ suite of libraries and programs.. BMC Evolutionary Biology.

[42] Chen K, Durand D, Farach-Colton M (2000). NOTUNG: a program for dating gene duplications and optimizing gene family trees.. Journal of Computational Biology.

[43] Durand D, Halldórsson BV, Vernot B (2006). A hybrid micro-macroevolutionary approach to gene tree reconstruction.. Journal of Computational Biology.

[44] James TY, Kauff F, Schoch CL, Matheny PB, Hofstetter V (2006). Reconstructing the early evolution of Fungi using a six-gene phylogeny.. Nature.

[45] Lewis PO, Holder MT, Holsinger KE (2005). Polytomies and Bayesian phylogenetic inference.. Systematic Biology.

[46] Hurst LD (2002). The Ka/Ks ratio: diagnosing the form of sequence evolution.. Trends in Genetics.

[47] Bielawski JP, Yang Z (2004). A maximum likelihood method for detecting functional divergence at individual codon sites, with application to gene family evolution.. Journal of Molecular Evolution.

[48] Bielawski J, Yang Z (2003). Maximum likelihood methods for detecting adaptive evolution after gene duplication.. Journal of Structural and Functional Genomics.

[49] Xia X, Xie Z (2001). DAMBE: software package for data analysis in molecular biology and evolution.. Journal of Heredity.

[50] Yang Z (2007). PAML 4: phylogenetic analysis by maximum likelihood.. Molecular Biology and Evolution.

[51] Suzuki Y, Nei M (2001). Reliabilities of parsimony-based and likelihood-based methods for detecting positive selection at single amino acid sites.. Molecular Biology and Evolution.

[52] Yang Z (1998). Likelihood ratio tests for detecting positive selection and application to primate lysozyme evolution.. Molecular Biology and Evolution.

[53] Yang Z, Wong WSW, Nielsen R (2005). Bayes empirical Bayes inference of amino acid sites under positive selection.. Molecular Biology and Evolution.

[54] Gillespie J (1991). The Causes of Molecular Evolution.

[55] Zhang J, Nielsen R, Yang Z (2005). Evaluation of an improved branch-site likelihood method for detecting positive selection at the molecular level.. Molecular Biology and Evolution.

[56] Nielsen R, Yang Z (1998). Likelihood models for detecting positively selected amino acid sites and applications to the HIV-1 envelope gene.. Genetics.

[57] Bonferroni C (1935). Il calcolo delle assicurazioni su gruppi di teste.. Studi in Onore del Professore Salvatore Ortu Carboni.

[58] Bonferroni CE (1936). Teoria statistica delle classi e calcolo delle probabilita.. Libreria internazionale Seeber.

[59] McAuley KE, Yao JX, Dodson EJ, Lehmbeck J, Ostergaard P (2001). A quick solution: ab initio structure determination of a 19 kDa metalloproteinase using ACORN.. Acta Crystallographica Section D: Biological Crystallography.

[60] Guex N, Peitsch MC (1997). SWISS-MODEL and the Swiss-pdb Viewer: an environment for comparative protein modeling.. Electrophoresis.

[61] Schwede T, Kopp J, Guex N, Peitsch MC (2003). SWISS-MODEL: an automated protein homology-modeling server.. Nucleic Acids Research.

[62] Arnold K, Bordoli L, Kopp J, Schwede T (2006). The SWISS-MODEL workspace: a web-based environment for protein structure homology modelling.. Bioinformatics.

[63] DeLano WL (2002). The PyMOL molecular graphics system.

[64] Yang Z (1997). PAML: a program package for phylogenetic analysis by maximum likelihood.. Comput Appl Biosci.

[65] Desjarlais JR, Handel TM (1999). Side-chain and backbone flexibility in protein core design.. Journal of Molecular Biology.

[66] Najmanovich R, Kuttner J, Sobolev V, Edelman M (2000). Side-chain flexibility in proteins upon ligand binding.. Proteins Structure Function and Genetics.

