# Supplementary figures and images for: Molecular Evolution of the Deuterolysin (M35) Family Genes in Coccidioides
Source: PLoS One. 2012 Feb 20;7(2):e31536. doi: 10.1371/journal.pone.0031536 (PMC3282736; doi:10.1371/journal.pone.0031536)

**Figure S1.** Whole protein alignment of the M35 genes with MUSCLE 3.5.


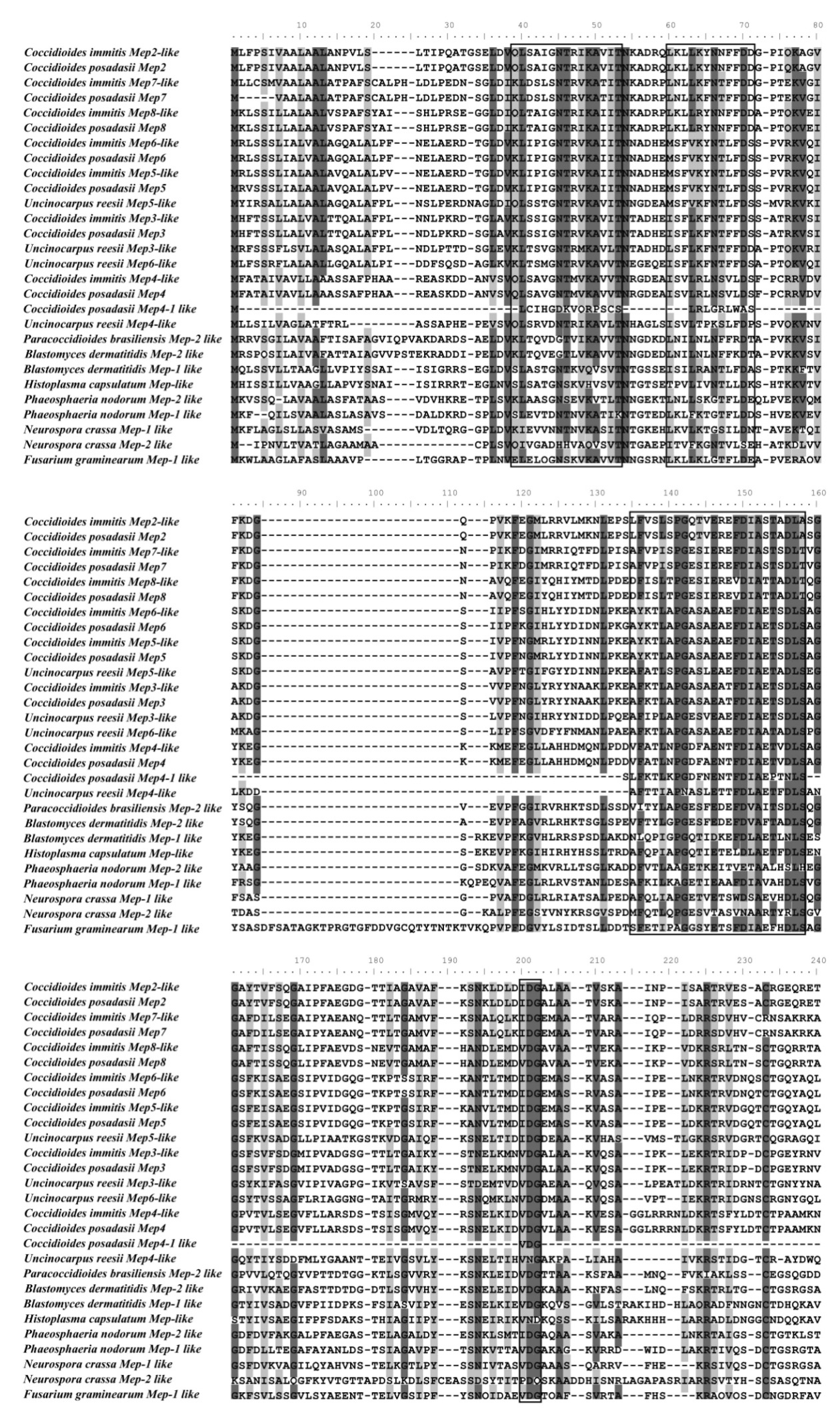


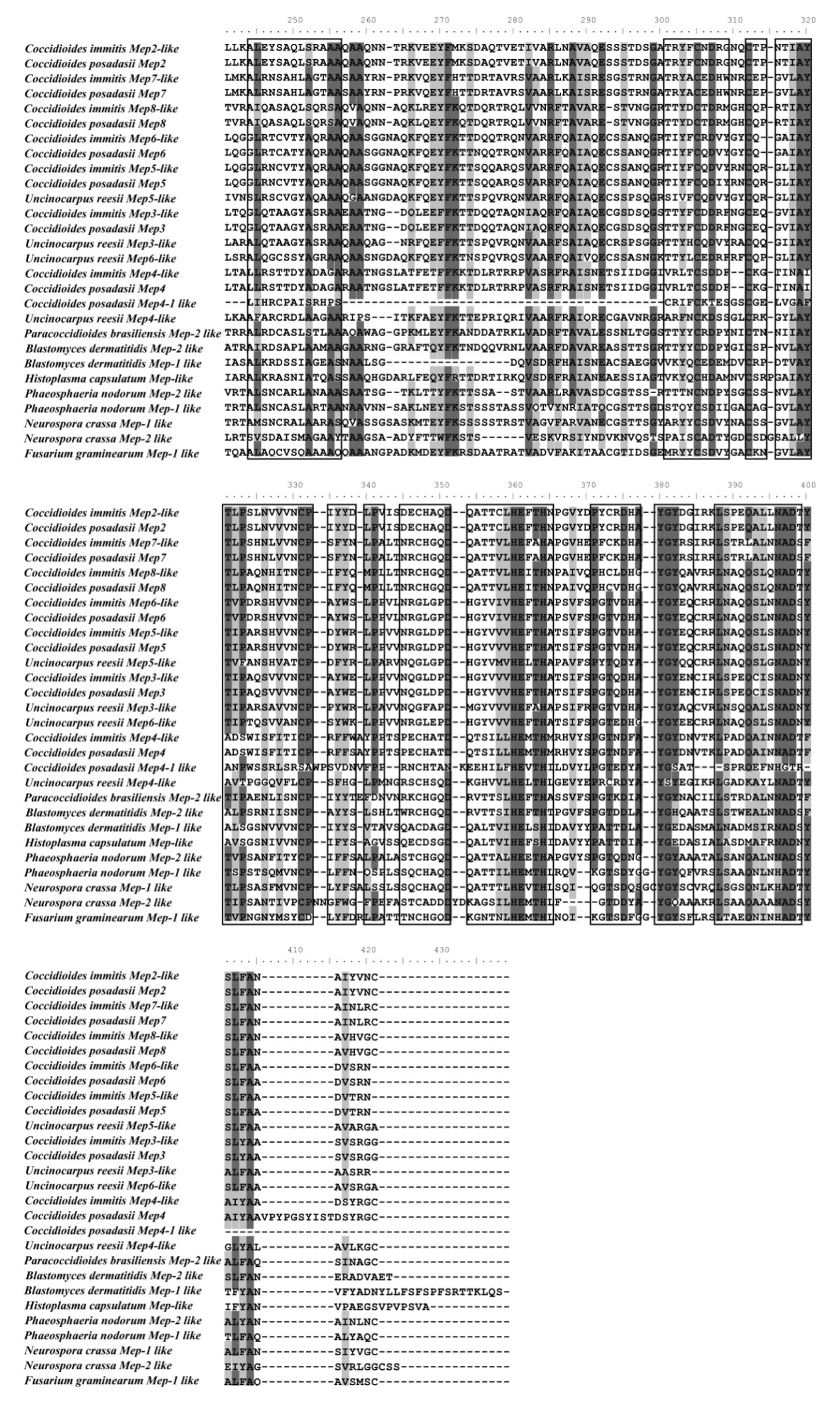

Supplement: Figure S1 — Whole protein alignment of the M35 genes with MUSCLE 3.5. Sequences were aligned using MUSCLE v3.5 with default settings [26]. 139 amino acids (corresponding to 417-bp nucleotide positions) obtained from PAL2NAL v13 [27] were encompassed by frame with black edge. (DOC) [file pone.0031536.s001.doc]

**Figure S2.** Protein alignment of M35 genes aligned with strategy 2.


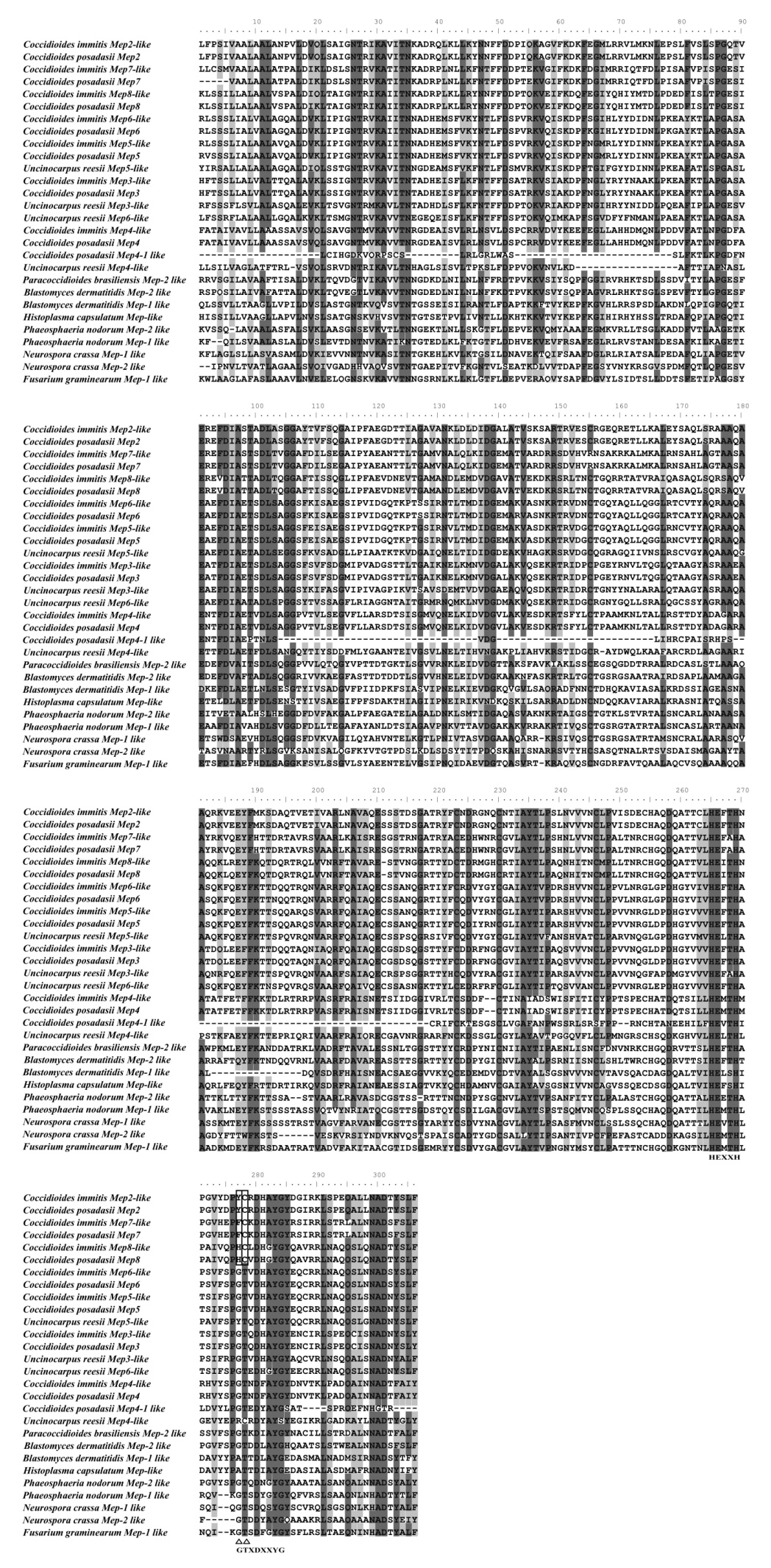

Supplement: Figure S2 — Protein alignment of M35 genes aligned with strategy 2. Sequences were aligned using MUSCLE v3.5 software with default settings [26]. The ambiguous areas of alignment were located and removed by using the program Gblocks 0.91b [28], [29] with default parameters. The gap selection criterion “with half” was used here. A 918-bp alignment was obtained. (DOC) [file pone.0031536.s002.doc]

**Figure S3.** Protein alignment of M35 genes aligned with strategy 3


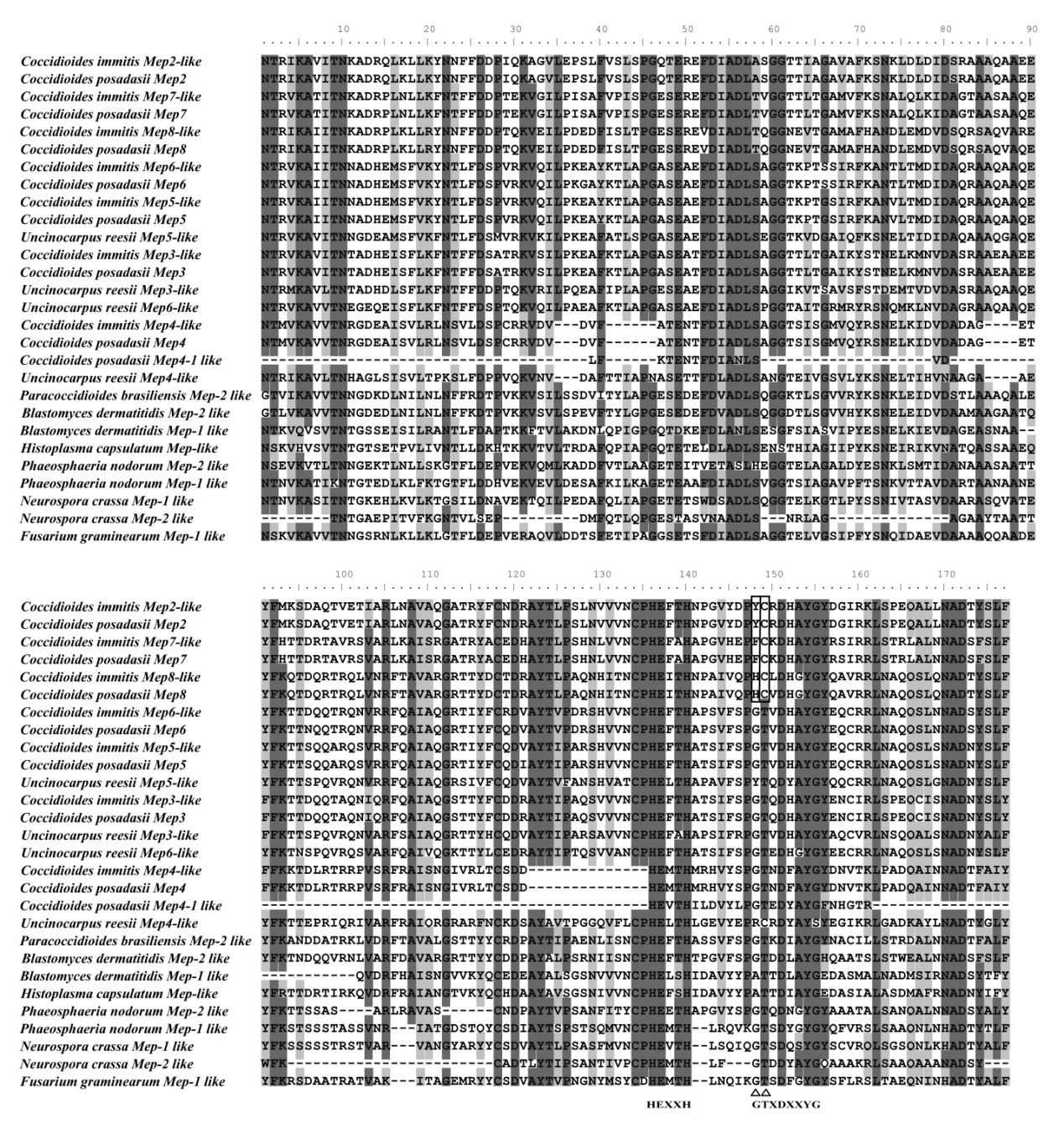

Supplement: Figure S3 — Protein alignment of M35 genes aligned with strategy 3. Sequences were aligned using PRANK with default settings [30], [31]. The ambiguous areas of alignment were located and removed by using the program Gblocks 0.91b [28], [29] with default parameters. The gap selection criterion “with half” was used here. A 531-bp alignment was obtained. (DOC) [file pone.0031536.s003.doc]

**Figure S4.** Domain compositions of M35 family genes analyzed with InterProScan.


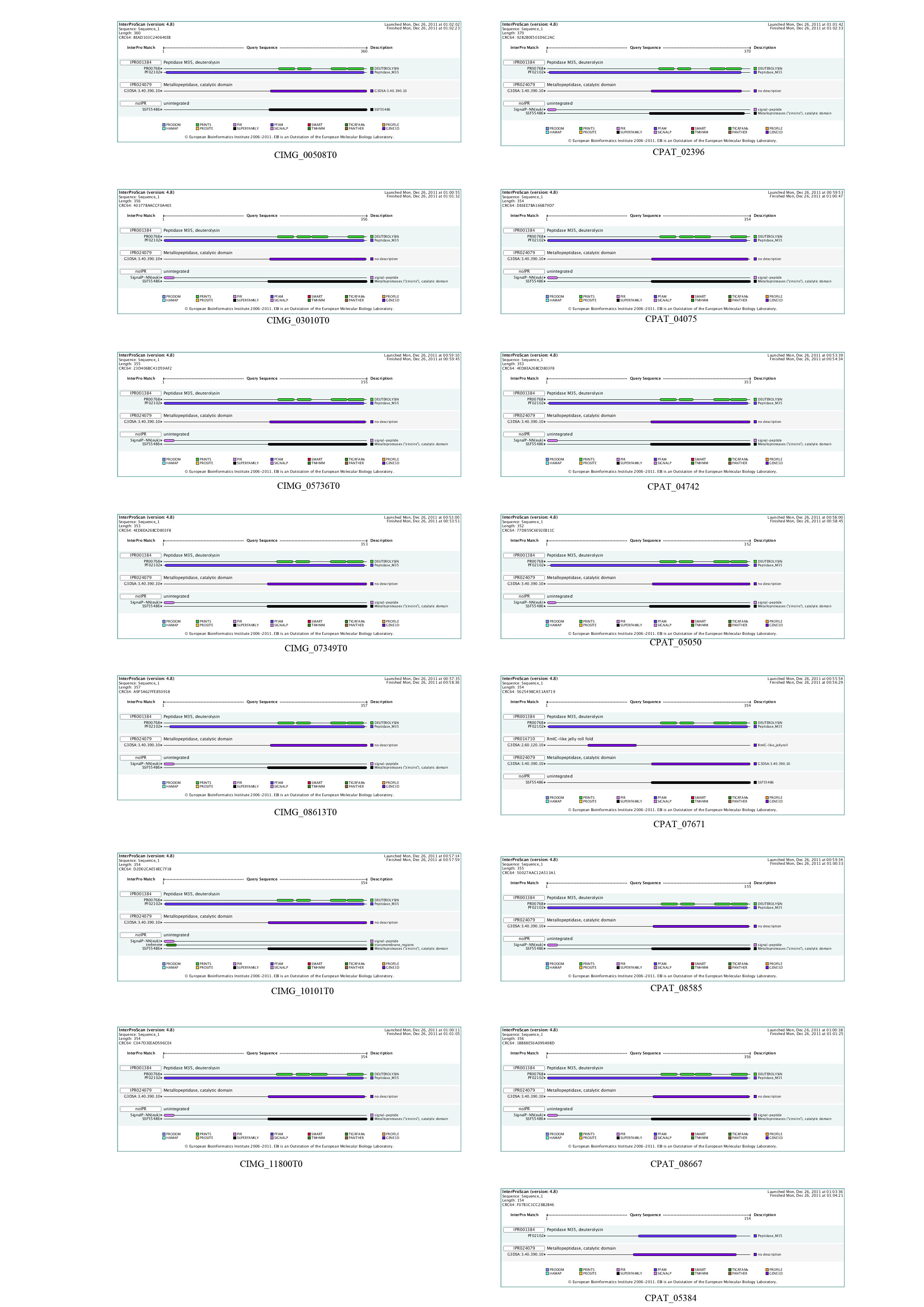


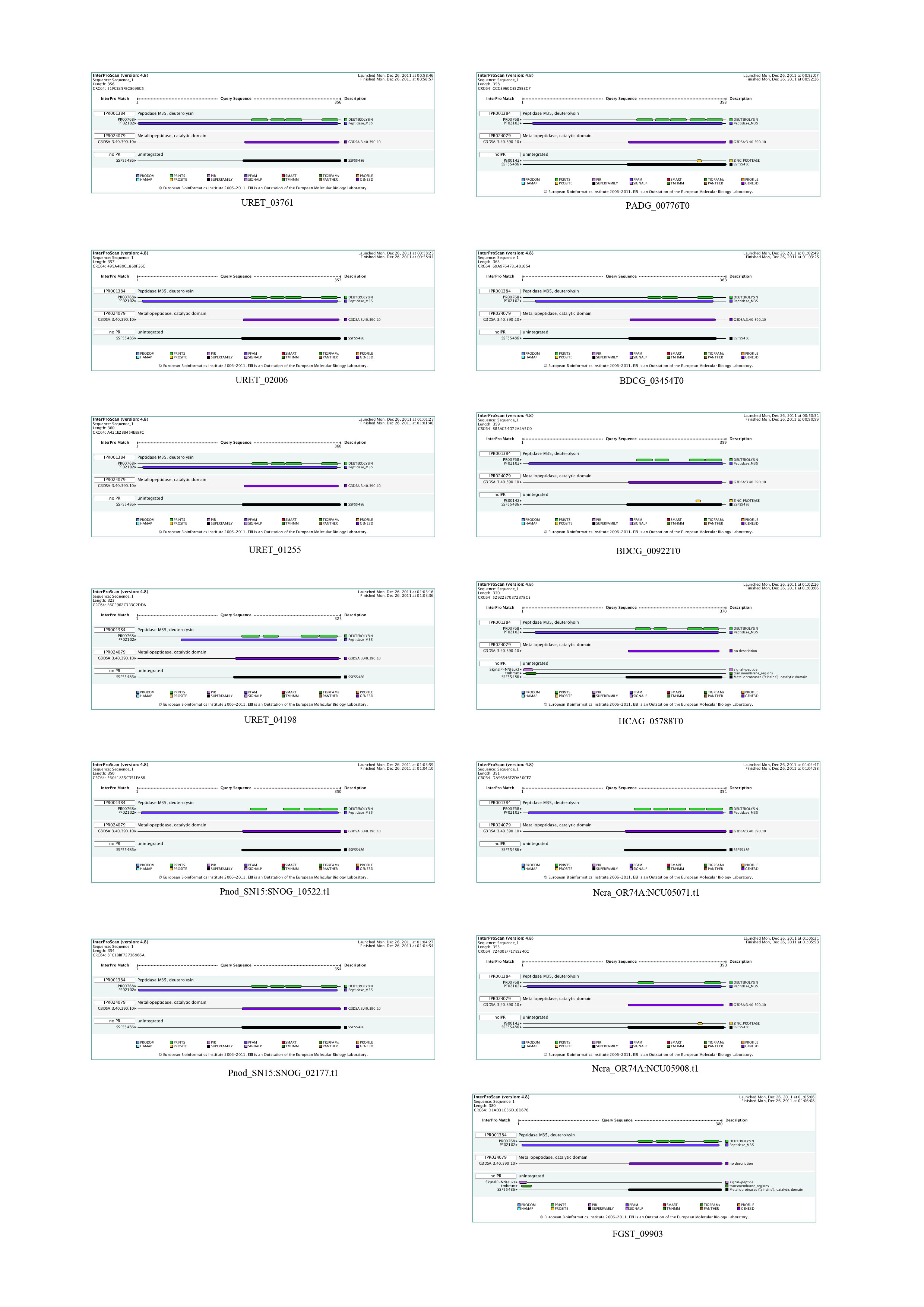

Supplement: Figure S4 — Domain compositions of M35 family genes analyzed with InterProScan. InterProScan (http://www.ebi.ac.uk/Tools/InterProScan/) [25] was used to analyze domain compositions of all the 28 M35 family genes. (DOC) [file pone.0031536.s004.doc]

**Figure S5.** Phylogenetic trees of M35 family genes based on the alignment strategy 2**.**


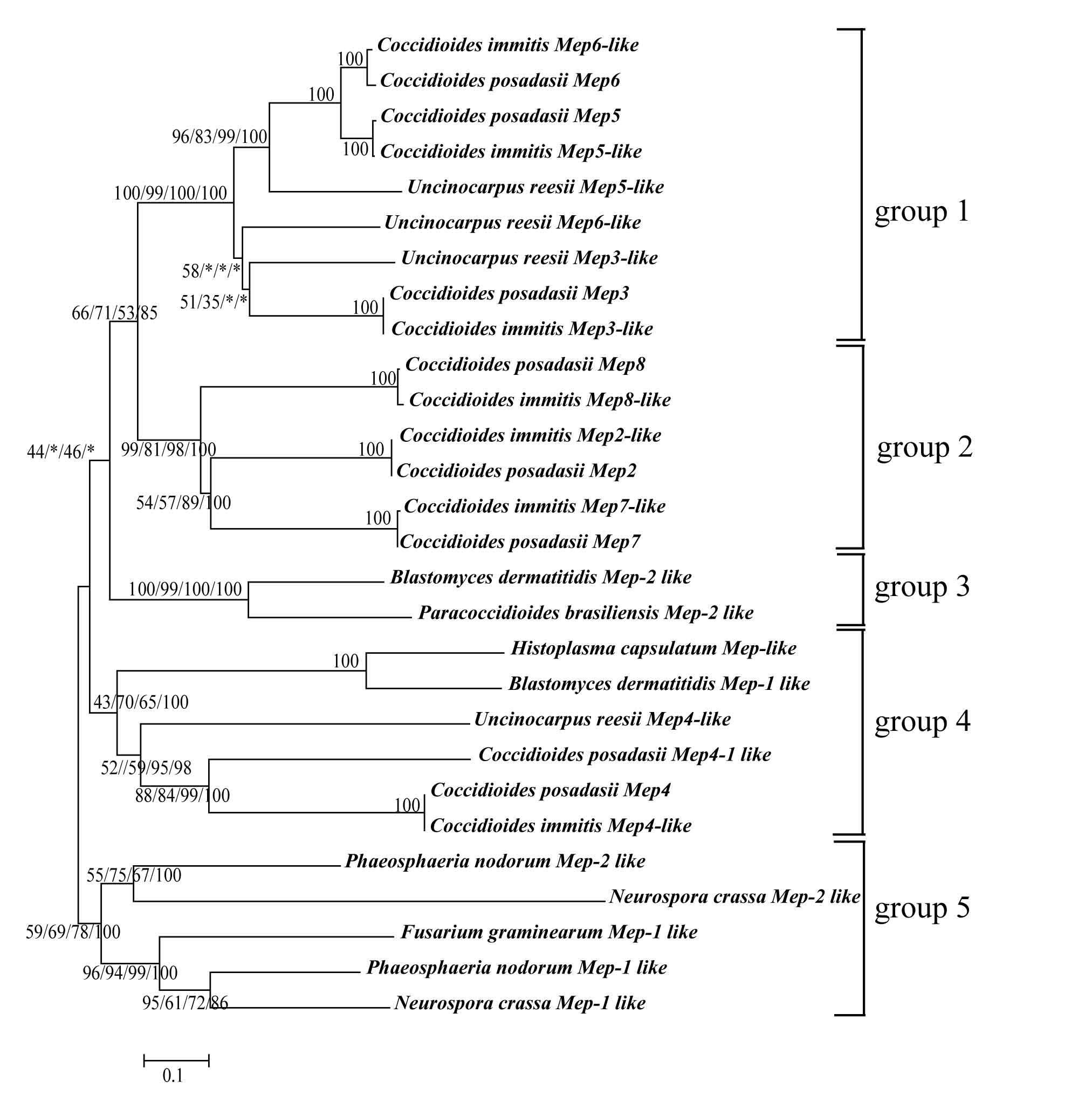

Supplement: Figure S5 — Phylogenetic trees of M35 family genes based on the alignment strategy 2. Support values for the topology obtained from four analyses are listed as percentages in the order A/B/C/D. A is the bootstrap support from NJ analysis. B is the posterior probability from PhyloBayes. C is the bootstrap from ML analysis and D is the posterior probability from MrBayes. The symbol (*) indicates the topological differences between different trees. (DOC) [file pone.0031536.s005.doc]

**Figure S6.** Phylogenetic trees of M35 family genes based on the alignment strategy 3**.**


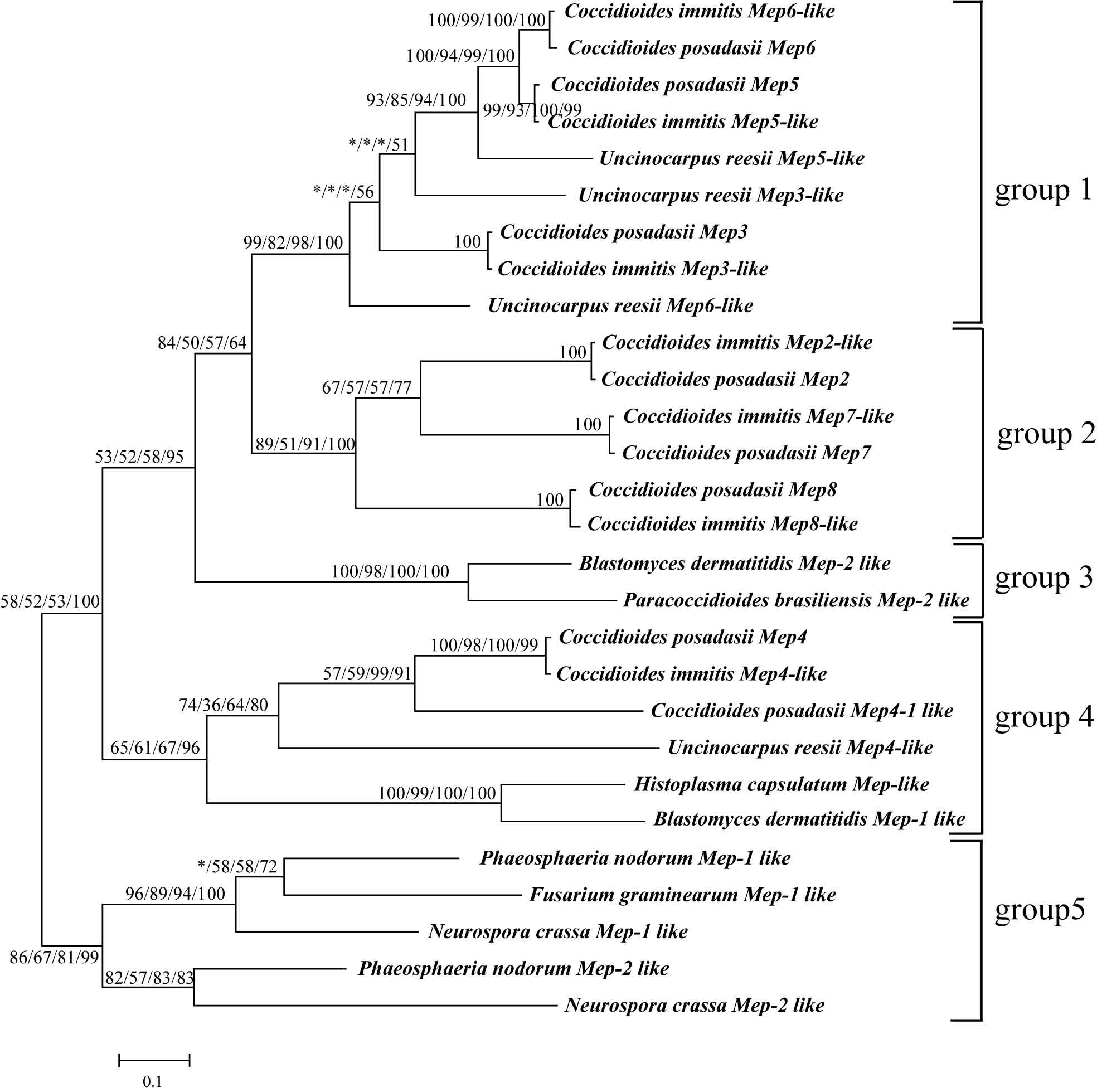

Supplement: Figure S6 — Phylogenetic trees of M35 family genes based on the alignment strategy 3. Support values for the topology obtained from four analyses are listed as percentages in the order A/B/C/D. A is the bootstrap support from NJ analysis. B is the posterior probability from PhyloBayes. C is the bootstrap from ML analysis and D is the posterior probability from MrBayes. The symbol (*) indicates the topological differences between different trees. (DOC) [file pone.0031536.s006.doc]

**Figure S7.** Plot of transitions/transversions versus genetic distance for M35 family genes.


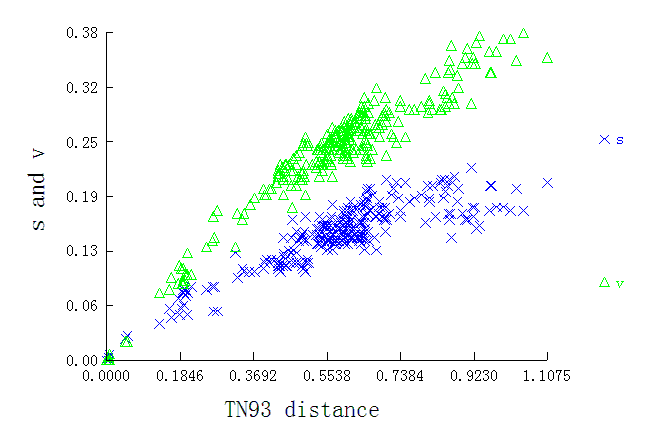

Supplement: Figure S7 — Plot of transitions/transversions versus genetic distance for M35 family genes. The estimated number of transitions (s) and transversions (v) for each pairwise comparison is plotted against the genetic distance (d) calculated with the TN93 model of nucleotide substitution using DAMBE [49]. (DOC) [file pone.0031536.s007.doc]
